# Highly Efficient SARS-CoV-2 Infection of Human Cardiomyocytes: Spike Protein-Mediated Cell Fusion and Its Inhibition

**DOI:** 10.1128/JVI.01368-21

**Published:** 2021-11-23

**Authors:** Chanakha K. Navaratnarajah, David R. Pease, Peter J. Halfmann, Biruhalem Taye, Alison Barkhymer, Kyle G. Howell, Jon E. Charlesworth, Trace A. Christensen, Yoshihiro Kawaoka, Roberto Cattaneo, Jay W. Schneider

**Affiliations:** a Department of Molecular Medicine, Mayo Clinic, Rochester, Minnesota, USA; b Discovery Engine/Program for Hypoplastic Left Heart Syndrome, Mayo Clinic, Rochester, Minnesota, USA; c Influenza Research Institute, Department of Pathobiological Sciences, School of Veterinary Medicine, University of Wisconsin-Madison, Madison, Wisconsin, USA; d Mayo Microscopy and Cell Analysis Core, Mayo Clinic, Rochester, Minnesota, USA; e Division of Virology, Department of Microbiology and Immunology, Institute of Medical Science, University of Tokyo, Tokyo, Japan; University of Kentucky College of Medicine

**Keywords:** cardiac tropism, cell-cell fusion, coronavirus, fusion inhibition, human cardiomyocyte

## Abstract

Severe cardiovascular complications can occur in coronavirus disease of 2019 (COVID-19) patients. Cardiac damage is attributed mostly to the aberrant host response to acute respiratory infection. However, direct infection of cardiac tissue by severe acute respiratory syndrome coronavirus 2 (SARS-CoV-2) also occurs. We examined here the cardiac tropism of SARS-CoV-2 in spontaneously beating human induced pluripotent stem cell-derived cardiomyocytes (hiPSC-CMs). These cardiomyocytes express the angiotensin-converting enzyme 2 (ACE2) receptor but not the transmembrane protease serine 2 (TMPRSS2) that mediates spike protein cleavage in the lungs. Nevertheless, SARS-CoV-2 infection of hiPSC-CMs was prolific; viral transcripts accounted for about 88% of total mRNA. In the cytoplasm of infected hiPSC-CMs, smooth-walled exocytic vesicles contained numerous 65- to 90-nm particles with canonical ribonucleocapsid structures, and virus-like particles with knob-like spikes covered the cell surface. To better understand how SARS-CoV-2 spreads in hiPSC-CMs, we engineered an expression vector coding for the spike protein with a monomeric emerald-green fluorescent protein fused to its cytoplasmic tail (S-mEm). Proteolytic processing of S-mEm and the parental spike were equivalent. Live cell imaging tracked spread of S-mEm cell-to-cell and documented formation of syncytia. A cell-permeable, peptide-based molecule that blocks the catalytic site of furin and furin-like proteases abolished cell fusion. A spike mutant with the single amino acid change R682S that disrupts the multibasic furin cleavage motif was fusion inactive. Thus, SARS-CoV-2 replicates efficiently in hiPSC-CMs and furin, and/or furin-like-protease activation of its spike protein is required for fusion-based cytopathology. This hiPSC-CM platform enables target-based drug discovery in cardiac COVID-19.

**IMPORTANCE** Cardiac complications frequently observed in COVID-19 patients are tentatively attributed to systemic inflammation and thrombosis, but viral replication has occasionally been confirmed in cardiac tissue autopsy materials. We developed an *in vitro* model of SARS-CoV-2 spread in myocardium using induced pluripotent stem cell-derived cardiomyocytes. In these highly differentiated cells, viral transcription levels exceeded those previously documented in permissive transformed cell lines. To better understand the mechanisms of SARS-CoV-2 spread, we expressed a fluorescent version of its spike protein that allowed us to characterize a fusion-based cytopathic effect. A mutant of the spike protein with a single amino acid mutation in the furin/furin-like protease cleavage site lost cytopathic function. Of note, the fusion activities of the spike protein of other coronaviruses correlated with the level of cardiovascular complications observed in infections with the respective viruses. These data indicate that SARS-CoV-2 may cause cardiac damage by fusing cardiomyocytes.

## INTRODUCTION

Severe acute respiratory syndrome coronavirus 2 (SARS-CoV-2), the coronavirus family member that most recently adapted to humans, is the etiologic agent of the coronavirus disease of 2019 (COVID-19). While the four coronaviruses endemic to humans (HCoV-229E, -NL63, -OC43, and -HKU1) impact mainly the respiratory tract and usually cause mild symptoms, SARS-CoV-2, like the other emerging coronaviruses, SARS-CoV and Middle East respiratory syndrome coronavirus (MERS-CoV), can cause lethal systemic symptoms (1).

Systemic consequences of the three emerging coronaviruses include cardiovascular complications. In particular, SARS-CoV-2 infection causes myocardial disease in a significant fraction of COVID-19 patients (2). Complications include worsening of preexisting conditions and the onset of new disorders (3–5). New disorders range from myocardial injury with or without classic coronary microvascular occlusion to arrhythmias and heart failure (3, 6).

Many cardiac symptoms have been tentatively attributed to aberrant host responses to acute respiratory infection (7, 8), but the complex mechanisms of cardiac disease are incompletely understood (9). As SARS-CoV-2 nucleic acids and proteins have been occasionally detected in cardiac tissue (10–18), productive SARS-CoV-2 infection of cardiomyocytes may directly injure this tissue, causing organ dysfunction. However, this hypothesis is difficult to verify experimentally. Rigorous analyses of cardiac tissue are restricted to rare endomyocardial biopsy or autopsy materials, and animal models to study cardiovascular complications of any coronavirus infection remain imperfect (1, 19).

To overcome these limitations, functionally differentiated human induced pluripotent stem cell-derived cardiomyocytes (hiPSC-CMs) have been used to model SARS-CoV-2 spread in cardiac tissue (20–24). Based on hiPSC-CMs from a developmental stage with peak expression of the SARS-CoV-2 receptor, angiotensin-converting enzyme 2 (ACE2), we have established a model of SARS-CoV-2 infection in which SARS-CoV-2 replicates more efficiently than previously described by others. Electron microscopy analyses documented large amounts of coronavirus particles both within exocytic vesicles and at the surface of infected hiPSC-CMs, which readily form syncytia. By expressing the spike protein of SARS-CoV-2, we have gained insights into the mechanisms of its functional activation by host enzymes in cardiomyocytes.

## RESULTS

### Expression of virus entry factors in cardiomyocytes.

**FIG 1:**
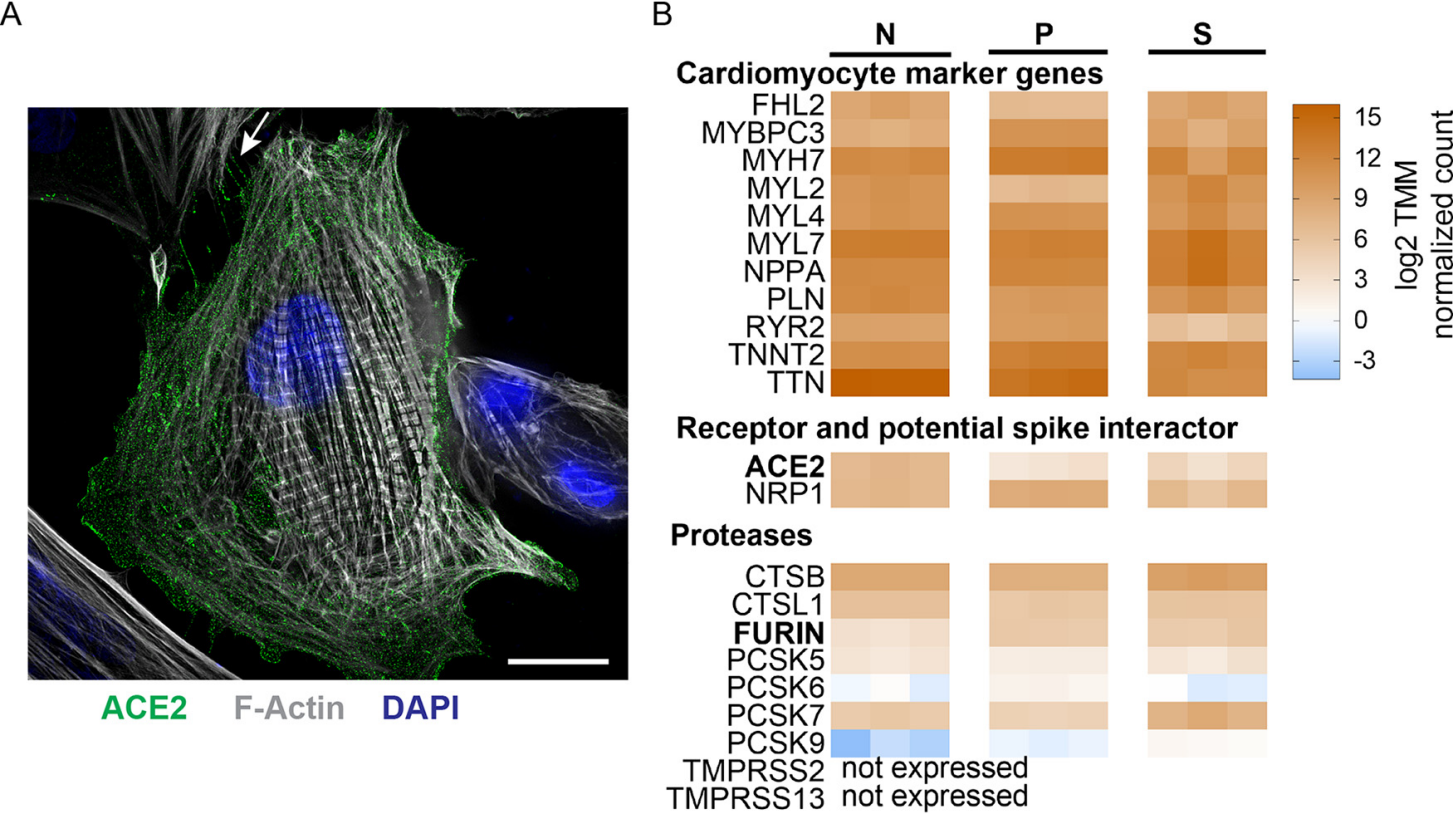
hiPSC-CMs express virus entry factors. (A) IF superresolution confocal microscopy analysis of ACE2 and F-actin expression in hiPSC-CMs. Scale bar, 10 μm. (B) Transcript levels of cardiomyocyte marker genes and of virus entry factors. Scale on the right, log_2_ trimmed mean of M values (TMM) normalized sequence read counts. N, this study; P, Perez-Bermejo et al. (22); S, Sharma et al. (20). Gene abbreviations: FHL2, four and a half LIM domains 2; MYBPC3, myosin binding protein C; MYH, myosin, heavy chain; MYL, myosin, light chain; NPPA, natriuretic peptide A; PLN, phospholamban; RYR2, ryanodine receptor 2; TNNT2, troponin T type 2; TTN, titin; ACE2, angiotensin-converting enzyme 2; NRP1, neuropilin 1, a potential spike interactor (64, 65); CTSB, cathepsin B; CTSL1, cathepsin L1; PCSK5, 6, 7, and 9, proprotein convertase subtilisin/kexin type 5, 6, 7, and 9; TMPRSS2 and 13, transmembrane serine protease 2 and 13.

We assessed whether ACE2, the SARS-CoV-2 receptor, and the spike-activating proteases transmembrane protease serine 2 (TMPRSS2) and cathepsin B (CTSB) are expressed during the differentiation process of human embryonic stem cells (hESC) into cardiomyocytes. The ACE2 transcription level peaked at day 20, those of cathepsin B remained stable, and TMPRSS2 transcripts were never detectable (see Table S1 in the supplemental material). Thus, we characterized our hiPSC-CMs (functionally equivalent to the hESC cardiomyocytes) at this differentiation stage. Super resolution immunofluorescence confocal microscopy documented cell surface expression of ACE2 and striated F-actin organization characteristic of contractile cardiomyocytes (Fig. 1A). In particular, ACE2 receptors clustered in raft-like puncta diffusely distributed across the sarcolemma (the cardiomyocyte plasma membrane) and extended into filopodia contacting adjacent cardiomyocytes (Fig. 1A, arrow highlights filopodia).

To further characterize day 20 differentiated cardiomyocytes, we analyzed their total cellular transcriptome by RNA sequencing (RNA-Seq). Cardiomyocyte differentiation markers were expressed in our hiPSC-CMs at levels similar to those documented in two other hiPSC-CM lines used for SARS-CoV-2 infection studies (Fig. 1B, compare panel N with panels P and S). Notably, the ACE2 receptor was expressed at higher levels in our cardiomyocytes than in those used in the other studies. In all three studies transcripts of the proteases, cathepsins B and L, furin, and four furin-like protein convertases (PCSK5, PCSK6, PCSK7, and PCSK9) were detected, but transcripts of the proteases that enable endosome-independent viral entry in the lungs and facilitate host-to-host transmission (TMPRSS2 and TMPRSS13) (25, 26) were below detection levels (less than 0.5 counts/million in at least 2 samples).

### Highly productive cardiomyocyte infection.

We used for our studies SARS-CoV-2/UW-001/Human/2020/Wisconsin (UW-001). This virus was isolated from a mild case in February 2020 and passaged in VeroE6 cells expressing TMPRSS2 to promote maintenance of the multibasic furin cleavage site (26, 27). Deep sequencing indicated that this isolate encodes D614 and did not accumulate any other mutations in the spike, particularly around the furin cleavage site (data not shown).

**FIG 2:**
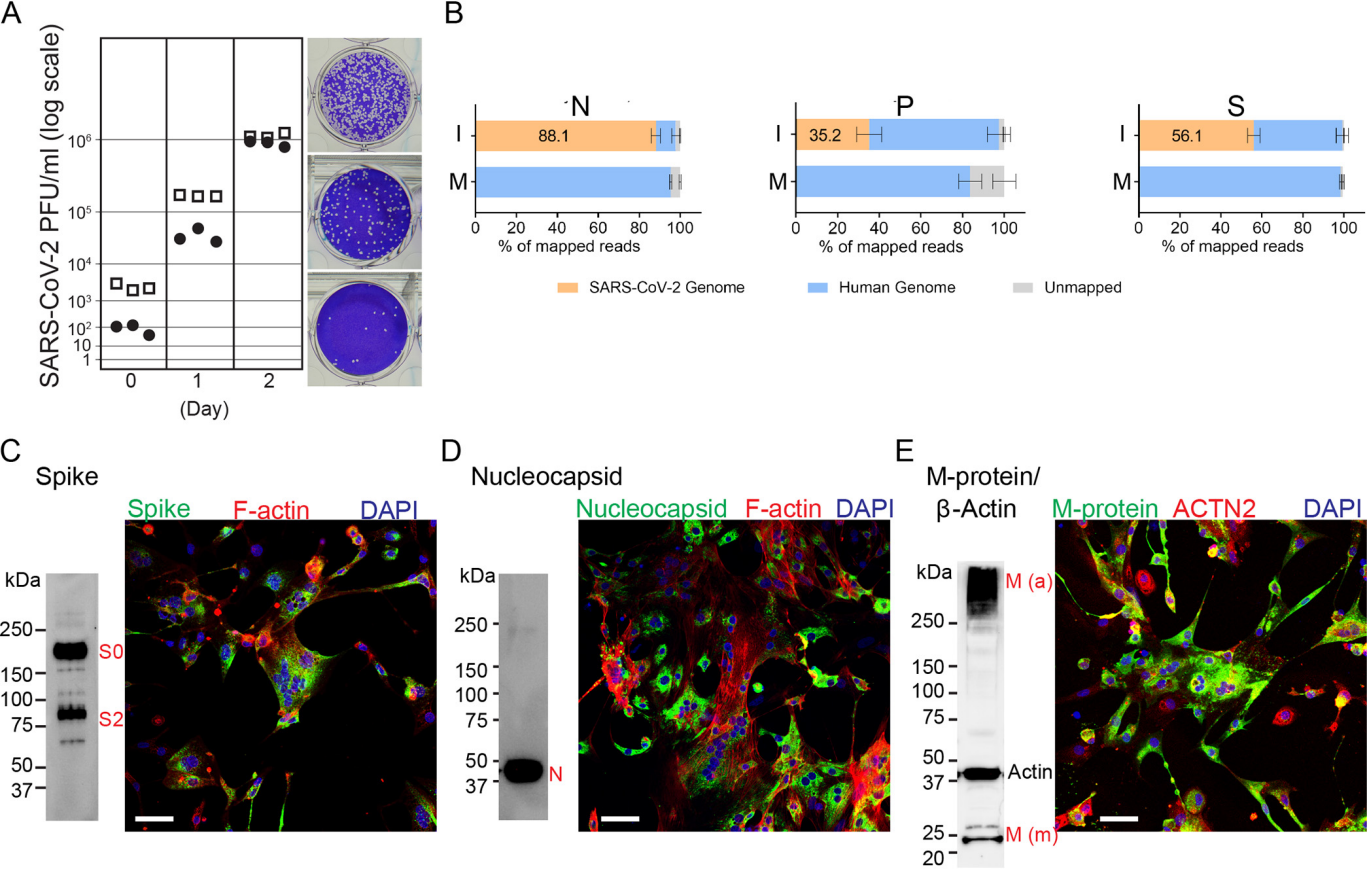
Efficient SARS-CoV-2 infection of hiPSC-CMs. (A) SARS-CoV-2 titers in two hiPSC-CM cell lines. Open squares, 81H4c2; filled dots, 47H4c45; each data point represents one biological replicate. (B) Quantification of viral transcripts in infected hiPSC-CMs from this study (N) and two published studies; P, Perez-Bermejo et al. (22); S, Sharma et al. (20). I, infected cardiomyocytes; M, mock-infected cardiomyocytes. (C to E) Companion immunoblots (left) and low-power IF confocal microscopy (right) of (C) SARS-CoV-2 spike glycoprotein (S0, S2), (D) nucleocapsid (N), and (E) membrane (M) protein, monomer (m) and insoluble aggregate (a) in hiPSC-CMs, 48 h postinfection. Scale bar, 50 μm.

We inoculated two independent lines of spontaneously beating hiPSC-CMs at a 0.01 multiplicity of infection (MOI) and monitored virus titer in the supernatant by plaque assay. Two days after inoculation, about 10^6^ infectious units/ml were produced (Fig. 2A). Strikingly, RNA-Seq analyses indicated that viral transcripts account for about 88% of the total cellular transcriptome (Fig. 2B, left panel). At the peak of SARS-CoV-2 infections of hiPSC-CM lines in two other studies, viral transcripts accounted for about 56% and 35% of the total cellular transcriptome (Fig. 2B, center and right panels).

We then sought to document expression, processing, and localization of the viral proteins. Immunoblot analyses of viral spike (S), nucleocapsid (N), and membrane (M) proteins confirmed high expression levels and accurate processing (Fig. 2C to E, left panels). Immunofluorescence (IF) microscopy indicated that all three proteins appear to be localized to the expected subcellular compartments (Fig. 2C to E, right panels). Taken together, these analyses confirmed highly productive infection of hiPSC-CMs by SARS-CoV-2.

### Abundant progeny virions in exocytic vesicles.

**FIG 3:**
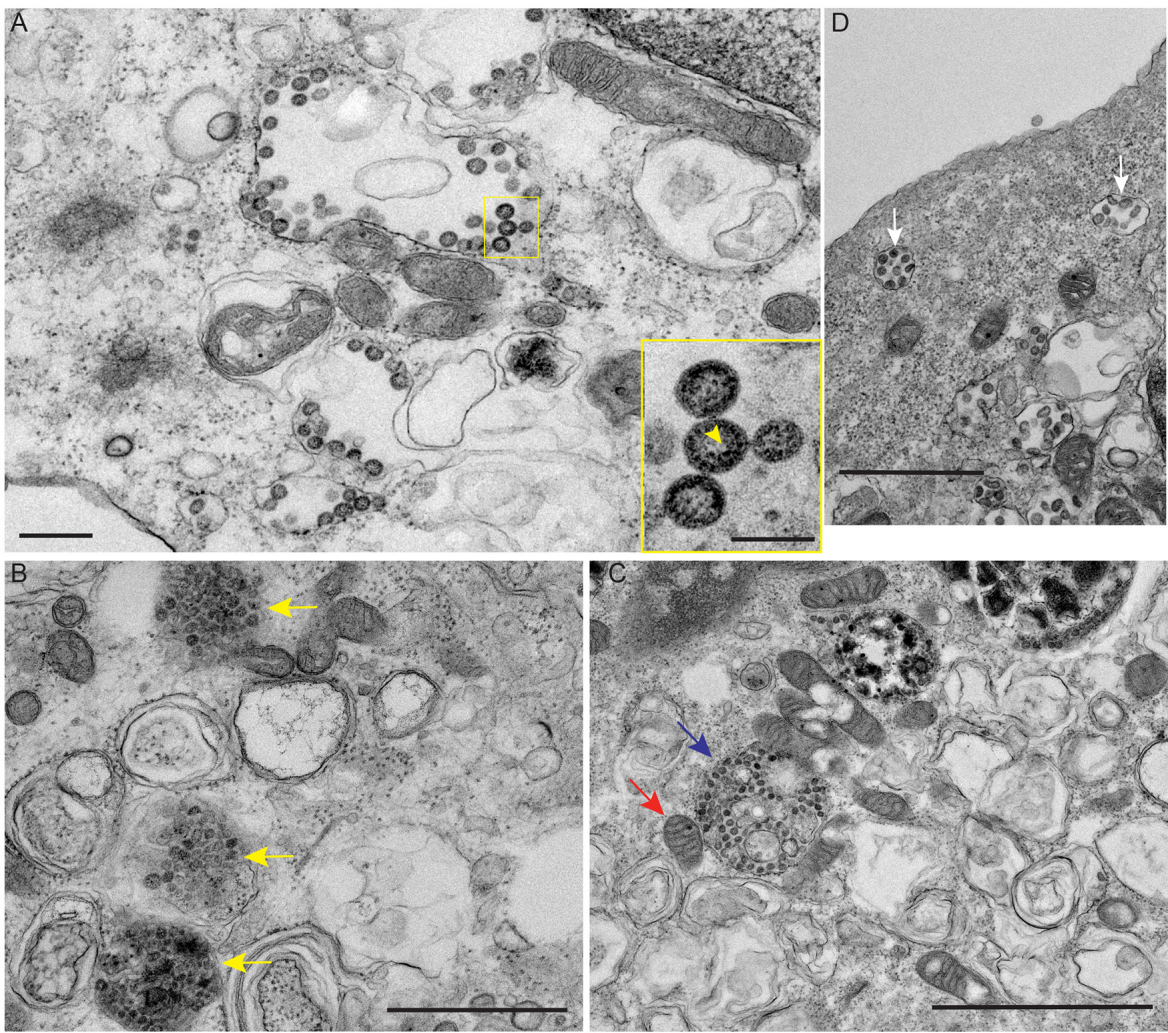
Maturation of SARS-CoV-2 in hiPSC-CMs. (A) TEM of SARS-CoV-2-infected hiPSC-CMs 48 h postinoculation revealing numerous SARS-CoV-2 particles concentrated within vesicles (yellow box). Scale bar, 400 nm. The inset panel is high magnification of the viral particles, demonstrating electron-dense ribonucleocapsid structures (yellow arrow). Scale bar, 100 nm. (B) SARS-CoV-2 clustered membranes (yellow arrows). Scale bar, 1 μm. (C) SARS-CoV-2 vesicle packet (blue arrow); mitochondria (red arrow). Scale bar, 2 μm. (D) SARS-CoV-2 exocytic vesicles (white arrows). Scale bar, 1 μm.

We then assessed by transmission electron microscopy (TEM) whether SARS-CoV-2 infection of hiPSC-CM recapitulated features characteristic of other coronavirus infections. TEM analyses revealed canonical double-membrane vesicles, endoplasmic reticulum-Golgi intermediate complex, and smooth-walled exocytic vesicles containing numerous 65- to 90-nm particles (Fig. 3A, yellow box). These are progeny virions with typical helical ribonucleocapsids surrounded by a membrane (Fig. 3A, inset). Other characteristic features of coronavirus infections detected in hiPSC-CMs included clustered membranes (Fig. 3B, yellow arrows), vesicle packets filled with virus particles (Fig. 3C, blue arrow), and exocytic vesicles filled with virus particles (Fig. 3D, white arrows). Thus, TEM analyses of infected hiPSC-CMs detected alterations of the cellular secretory pathway characteristic of coronavirus infections.

### Virus particles with knob-like spikes on the cardiomyocyte surface.

**FIG 4:**
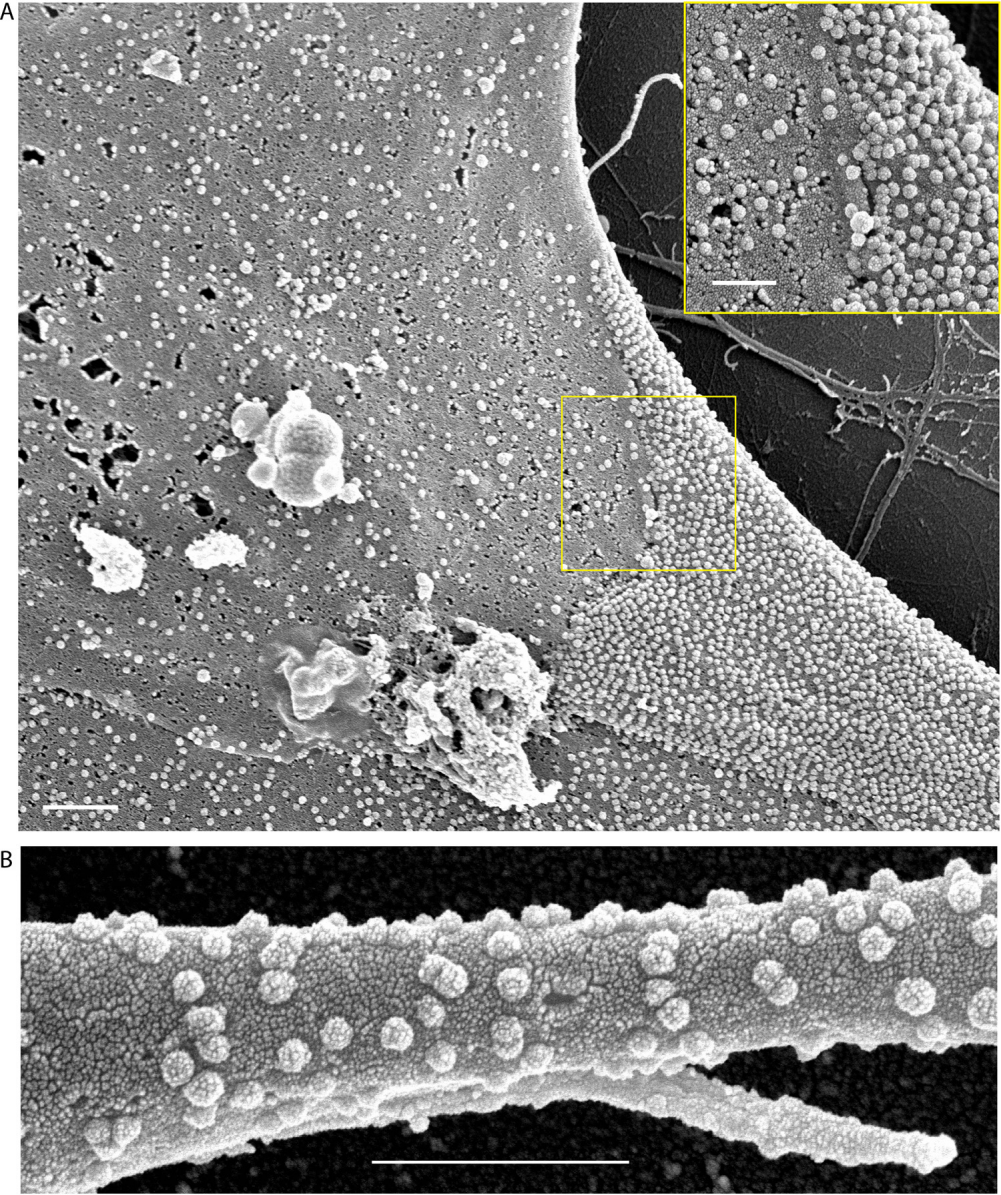
Surface of hiPSC-CMs infected with SARS-CoV-2. (A) Scale bar, 1 μm. The inset shows high magnification of the surface region within the yellow box. Scale bar, 500 nm. (B) High magnification SEM of hiPSC-CM filopodia dotted with SARS-CoV-2 viral particles. Scale bar, 1 μm.

We assessed whether typical SARS-CoV-2 particles are present on the surface of hiPSC-CMs by scanning electron microscopy (SEM), which revealed numerous particles on the plasma membrane. Fig. 4A shows an hiPSC-CM heavily carpeted with SARS-CoV-2 particles (rightmost cell) contacting two less heavily carpeted hiPSC-CMs at the upper and lower left with boundaries clearly demarcated, creating a patchwork mosaic. The inset magnifies the boundary highlighted by the yellow box. Viral particles cover the entire surface of the hiPSC-CM, including pseudo- and filopodia and show typical knob-like spikes (Fig. 4B). Thus, SEM analyses detected abundant bona fide virus particles on the hiPSC-CM surface, and individual cells produced different amounts of virus particles.

### Cytopathic effects and fusion of infected cardiomyocytes.

**FIG 5:**
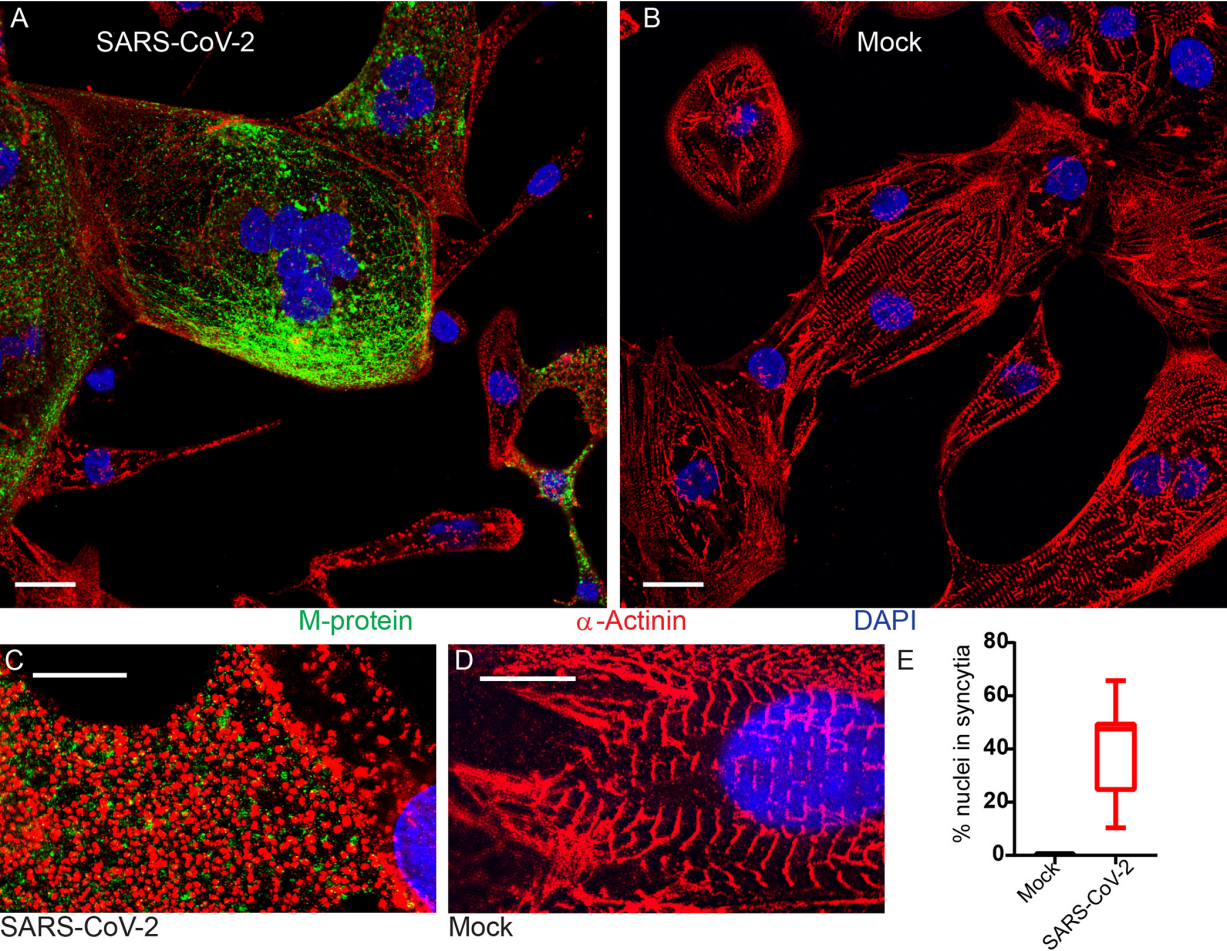
Cytopathic effects of SARS-CoV-2 in hiPSC-CMs. (A and B) IF confocal microscopy of SARS-CoV-2-infected (48 h postinfection) or mock-infected hiPSC-CMs, respectively. Scale bar, 20 μm. (C and D) IF superresolution confocal microscopy of infected and mock-infected hiPSC-CMs, respectively. Scale bars, 10 μm. (E) Quantification of cell fusion in SARS-CoV-2-infected and mock-infected hiPSC-CMs 48 h postinoculation (*n* = 3 biological replicates). “% nuclei” in syncytia denotes the percentage of total nuclei within syncytia. Box and whisker plots show median, upper, and lower quartiles and extremes; 12image fields were counted per condition with an average of 44 cells per image field.

We also monitored the cytopathic effects of SARS-CoV-2 infection of hiPSC-CMs by IF confocal microscopy. In Fig. 5A the nuclei of infected cells were stained with DAPI (blue), and the viral M protein and cytoskeletal α-actinin were stained with specific antibodies (green and red, respectively). Fig. 5B shows the same analyses on control uninfected hiPSC-CMs. In Fig. 5A giant cells with central clusters of nuclei, typical viral-mediated syncytia, were documented. These M-protein positive hiPSC-CMs demonstrated sarcomeric disassembly/fragmentation shown by disintegration of α-actinin Z-discs into randomly distributed puncta (Fig. 5C). Neither syncytium formation nor cytoskeletal disassembly were observed in mock-infected cells (Fig. 5D).

To quantify SARS-CoV-2-mediated hiPSC-CM fusion, α-actinin and SARS-CoV-2 M protein colabeled cells were imaged by IF confocal microscopy, and the percentage of nuclei found within syncytia was determined. In infected cells about half of the nuclei were found within syncytia, while in mock-infected cells less than 1% of the nuclei were found within syncytia (Fig. 5E). Thus, many infected cardiomyocytes fuse, and cytoskeletal disintegration may favor syncytium formation.

### A fluorescent viral spike protein fuses cardiomyocytes.

**FIG 6:**
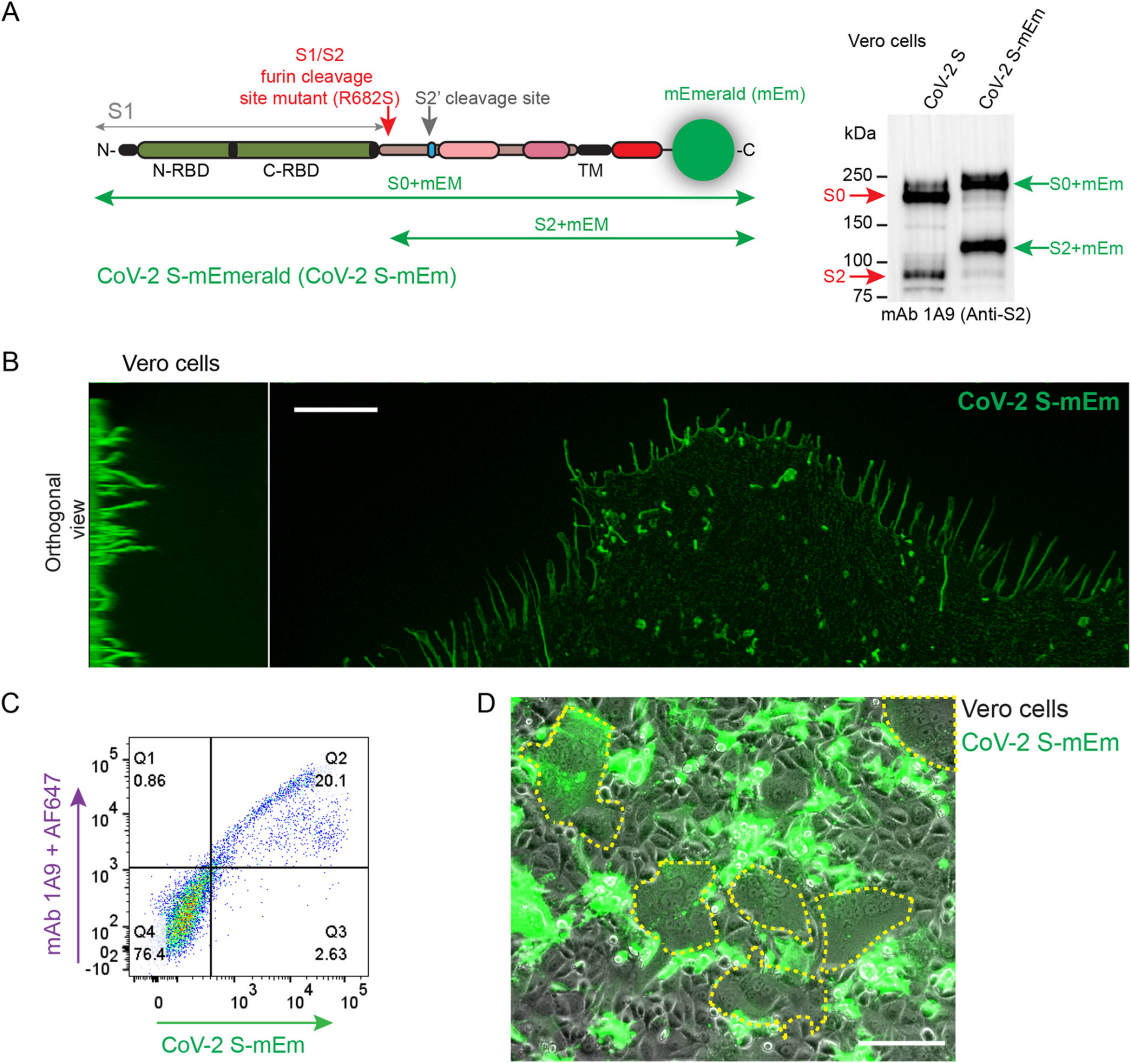
Expression and function of SARS CoV-2 spike protein tagged with mEmerald. (A) (Left) Schematic of SARS-CoV-2 S tagged with mEmerald (mEm) at the cytoplasmic tail. Cleavage at the S1/S2 furin site primes the spike protein for activation, while cleavage at the S2′ site (TMPRSS2/cathepsins) is required for fusion function. S1, S1 subunit; S2, S2 subunit; N-/C-RBD, N-/C-terminal receptor binding domains; TM, transmembrane segment. The fusion peptide is shown in blue, and heptad repeat 1 and 2, in magenta and dark magenta, respectively. The location of the furin cleavage mutant, R682S, is indicated. The monoclonal antibody 1A9, which was used to detect the spike proteins, binds to an exposed loop located close to heptad repeat 2. (Right) Immunoblot of the CoV-2 S and CoV-2 S-mEm proteins detecting their S0 and S2 subunits. (B) Superresolution confocal microscopy of CoV-2 S-mEM localization to Vero cell filopodia. Scale bar, 5 μm. (C) Cellular localization of the tagged spike protein in nonpermeabilized HeLa cells transfected with the expression plasmid for S-mEm. This protein was detected either by fluorescence emission (horizontal axis) or by using spike-specific-MAb 1A9 and AF647 conjugated secondary antibody (vertical axis). (D) Syncytia in Vero cells transfected with CoV-2 S-mEm are indicated by a dotted yellow line. Scale bar, 50 μm.

To characterize the mechanism of cell fusion, we engineered a SARS-CoV-2 full-length recombinant spike protein fused to modified emerald green fluorescent protein at its carboxyl terminus (CoV-2 S-mEm) (Fig. 6A, left panel). We validated this reagent in Vero cells that, like hiPSC-CMs, express ACE2 but not TMPRSS2. In these cells CoV-2 S-mEm was appropriately cleaved (Fig. 6A, right panel). Super resolution confocal microscopy localized CoV-2 S-mEm to hair-like plasma membrane extensions (Fig. 6B). Fluorescent activated cell sorting confirmed CoV-2 S-mEm cell surface expression (Fig. 6C). Live cell imaging tracked spread of the CoV-2 S-mEm signal from cell to cell through membrane fusion, generating syncytia (Fig. 6D).

**FIG 7:**
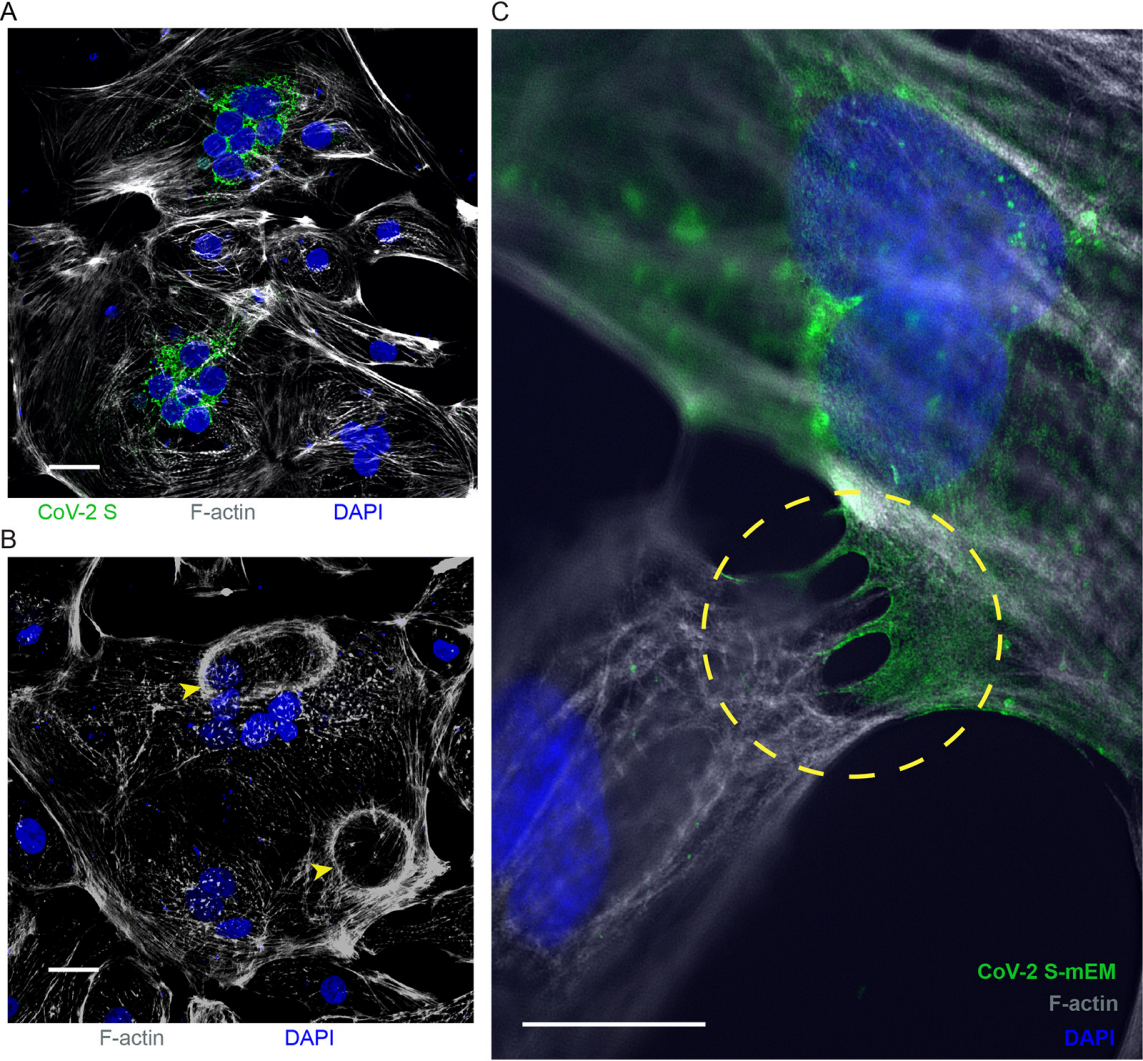
SARS-CoV-2 spike protein induces syncytia in hiPSC-CMs. (A) IF confocal microscopy of SARS-CoV-2 spike (CoV-2 S)-expressing hiPSC-CMs. Scale bar, 50 μm. The viral and cellular components visualized are indicated with their corresponding color below each panel. (B) IF confocal microscopy of CoV-2 S-expressing hiPSC-CM with enucleated actin cytoskeletal “corpses” (yellow arrows). (C) Superresolution confocal microscopy of CoV-2 S-mEM localization to hiPSC-CM filopodia directly contacting the sarcolemma of an adjacent hiPSC-CM (yellow circle). Scale bar, 2 μm.

We then assessed whether CoV-2 S-mEm fuses cardiomyocytes. Despite overall transfection efficiency of <5%, CoV-2 S-mEM expressing hiPSC-CMs produced syncytia with nuclei frequently arranged in clusters or rosettes (Fig. 7A). Some syncytia were characterized by circular or oval enucleated cytoskeletal “corpses” shown by F-actin phalloidin staining (Fig. 7B, yellow arrows). Super resolution confocal microscopy demonstrated fluorescent signal at the tips of dynamic pseudo- and filopodia contacting neighboring hiPSC-CMs (Fig. 7C, circle). Since hiPSC-CMs do not express TMPRSS2, we conclude that in these cells another protease must activate the SARS-CoV-2 spike.

### Furin activation of spike is required for cardiomyocyte fusion.

Knowing that furin, a protease located in the *trans*-Golgi apparatus that contributes to SARS-CoV-2 spike activation, is expressed in hiPSC-CM (Fig. 1B), we sought to block its function biochemically and genetically. For biochemical interference we used decanoyl-RVKR-CMK (furin inhibitor, FI), a cell-permeable peptide-based molecule that irreversibly blocks the catalytic site of furin and furin-like proteases. For genetic interference, we generated an expression vector differing from CoV-2 S through the single amino acid change R682S in order to disrupt the mutibasic furin cleavage site (28) (Fig. 6A, left panel).

**FIG 8:**
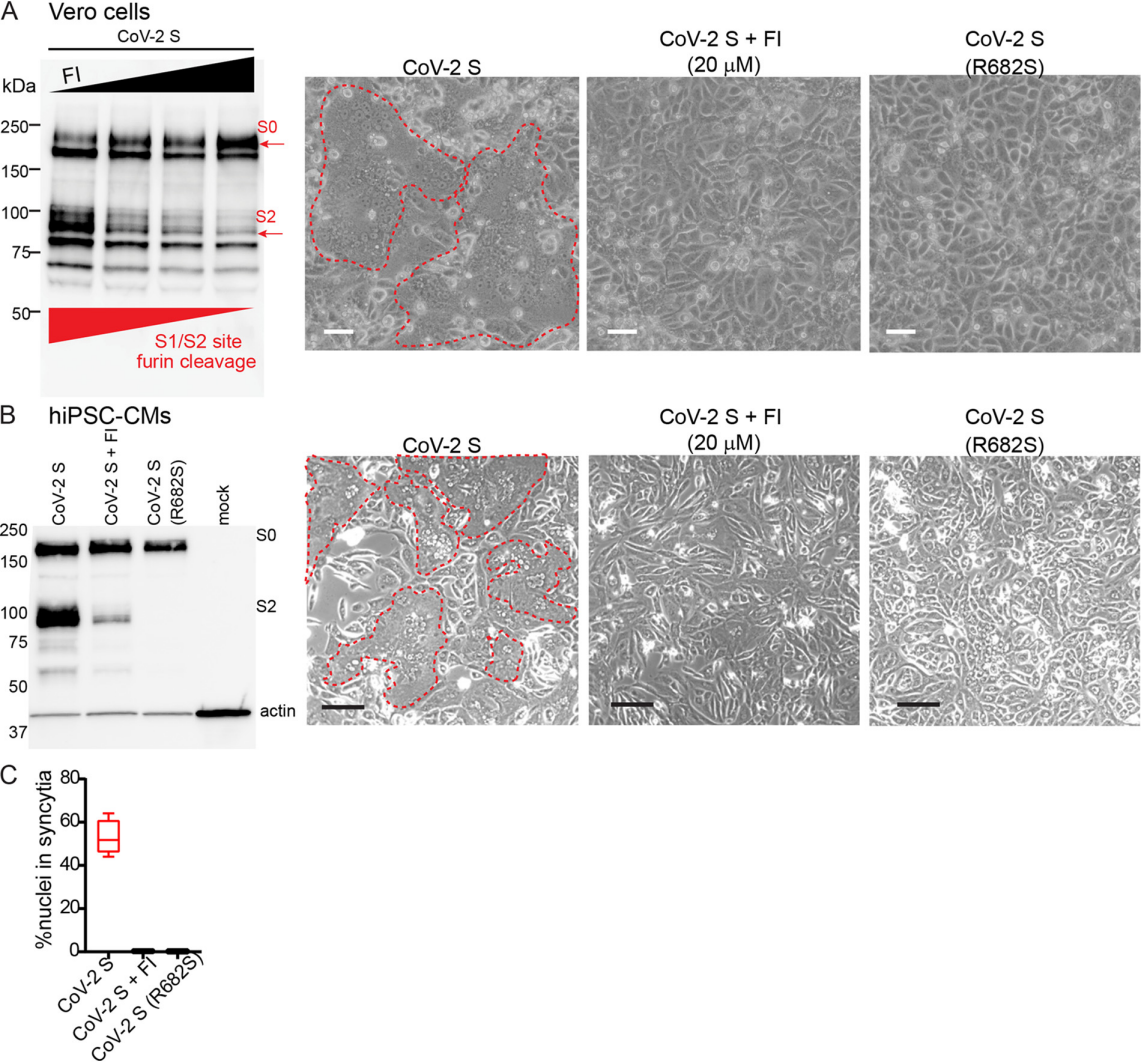
SARS-CoV-2 spike-generated syncytia are blocked by a furin inhibitor or a furin-cleavage mutant. (A) (Left panel) Immunoblot analysis of CoV-2 S protein processing (S0 cleavage into S1 and S2) in Vero cells treated with increasing concentrations of FI (0 μM, 5 μM, 10 μM, and 20 μM); cell lysates were separated by 4 to 15% SDS-PAGE under reducing conditions. The 2nd to 4th panels show phase contrast images of Vero cells expressing CoV-2 S in the absence or presence of 20 μM FI or of Vero cells expressing the R682S cleavage mutant, respectively, 72-h after transfection. Syncytia are demarcated by a dashed red line. Scale bar, 100 μm. (B) (Left panel) Gel analysis of CoV-2 S protein processing in the absence or presence of FI (20 μM) and of the processing of the CoV-2 S R682s furin cleavage mutant (R682S). Lysates of hiPSC-CM were separated by 4 to 15% SDS-PAGE under reducing conditions. The 2nd to 4th panels show phase contrast images of hiPSC-CM expressing CoV-2 S in the absence or presence of 20 μM FI or of Vero cells expressing the R682S cleavage mutant, respectively, 72-h after transfection. Syncytia are demarcated by a dashed red line. Scale bar, 100 μm. (C) Quantification of hiPSC-CM fusion. “% nuclei” in syncytia denotes the percentage of total nuclei within syncytia 48 h posttransfection (*n* = 3 biological replicates, *P* < 0.0001, ANOVA). Box and whisker plots for all quantification in this figure shows median, upper, and lower quartiles and extremes.

We validated these approaches in Vero cells. The left panel of Fig. 8A documents progressive inhibition of CoV-2 S protein processing (S0 cleavage into S1 and S2) by increasing concentrations of FI. The second and third panels show that fusion occurs in cells expressing CoV-2 S in the absence of FI, but not in its presence. The last panel shows that the R682S mutant of CoV-2 S is fusion-inactive.

Figure 8B shows that furin/furin-like protease activation of spike is required also for hiPSC-CM fusion. The left panel documents strong inhibition of spike protein processing by a high concentration of FI and complete lack of processing of the R682S mutant. The other panels show that FI or the mutant inhibits fusion of cardiomyocytes. Fig. 8C shows a quantitative analysis of hiPSC-CM fusion documenting approximately 99% inhibition by FI and by the mutation. Thus, expression of furin-activated SARS-CoV-2 spike protein in hiPSC-CM causes cell fusion that can be corrected pharmacologically.

### The MERS-CoV spike drives cardiomyocyte fusion with slow kinetics.

**FIG 9:**
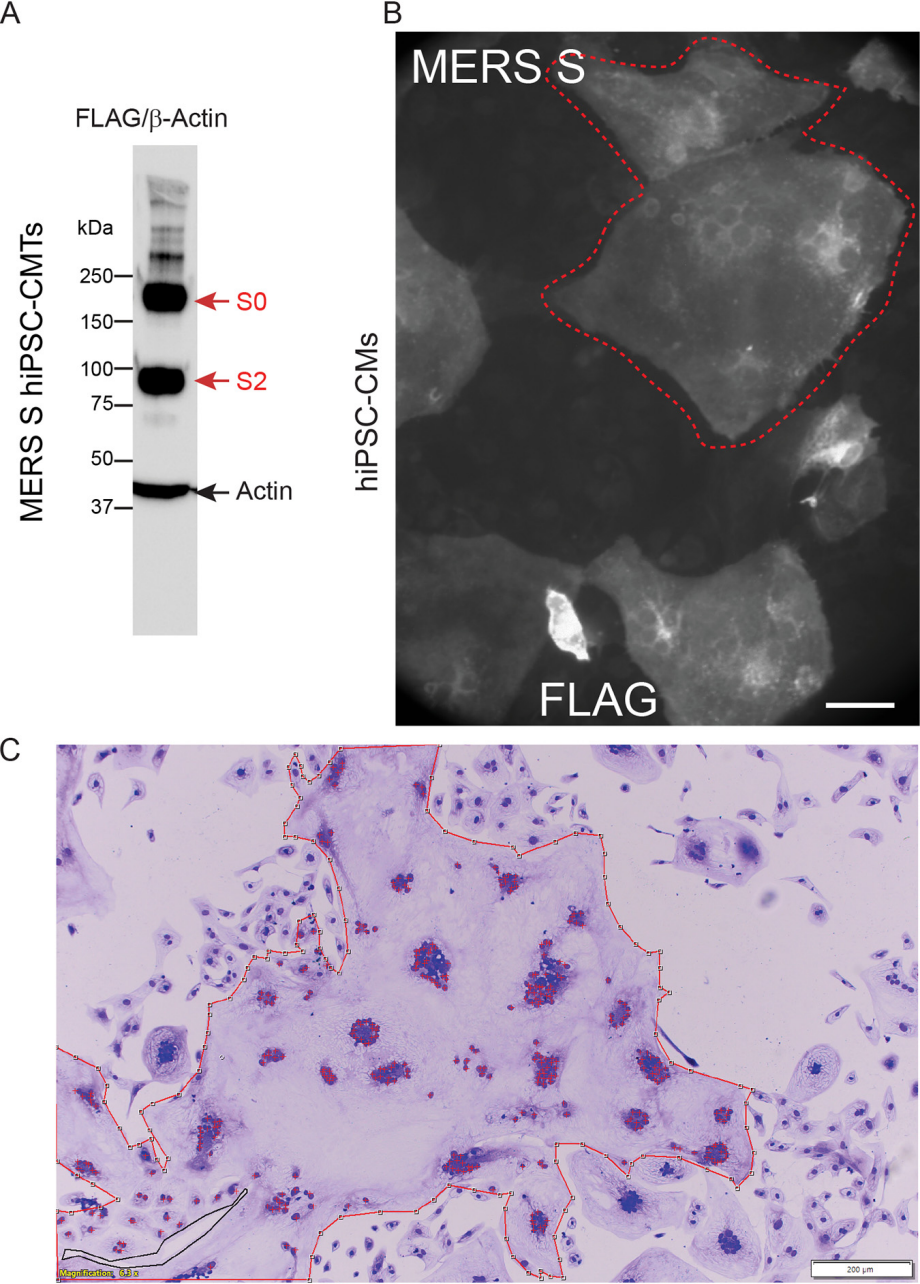
MERS spike-mediated syncytia. (A) Immunoblot of hiPSC-CM expressing recombinant MERS spike protein showing processing. The MERS spike S0 precursor and S2 cleaved subunit are detected through a FLAG epitope fused to the C terminus. High-molecular-weight (>250 kDa) oligomers, including trimers, are detected. (B) Anti-FLAG IF microscopy of MERS spike protein-mediated hiPSC-CM syncytia; the largest example is circled in red. Scale bar, 50 μm. (C) Bright-field microscopy of crystal violet-stained hiPSC-CM expressing recombinant MERS spike protein at 5 days posttransfection. A composite syncytium is circled in red. Scale bar, 200 μm.

Since SARS-CoV (29) and MERS-CoV (30) can cause cardiovascular complications, we asked whether their spike proteins can fuse hiPSC-CMs. As a negative control we used the spike protein of the common cold coronavirus HCoV-229E. Figure 9A shows correct processing of the MERS-CoV spike protein, and Fig. 9B and C demonstrate that this protein induces syncytium formation. Comparative analyses indicated that the MERS spike protein drove syncytium production with slower kinetics than the SARS-CoV-2 spike, while the spike proteins of SARS-CoV and of the common cold coronavirus HCoV-229E were inactive (data not shown). These levels of fusion activity correlate with the amounts of cardiovascular complications observed in infections with the respective viruses.

## DISCUSSION

Viruses can cause myocarditis and cardiomyopathies, but the mechanisms of disease are difficult to characterize experimentally (31, 32). The cardiac complications frequently observed in COVID-19 patients are tentatively attributed to aberrant host responses to acute respiratory infection, but SARS-CoV-2 replication has occasionally been confirmed in endomyocardial biopsy and autopsy cardiac tissue. While animal models to study SARS-CoV-2 infections of the heart are being developed, we have characterized virus spread in hiPSC-CMs. Infection of these highly differentiated cells was unexpectedly efficient, with the virus taking over almost 90% of the cellular transcriptome. SARS-CoV-2 infection reshaped subcellular morphologies (33, 34), secretory vesicles were filled with viral progeny, and virus particles with knob-like spikes carpeted the cardiomyocyte surface.

Human iPSC-CMs are permissive to SARS-CoV-2 infection. Two previous studies documented that viral transcript accounts for up to 35% or 55% of the hiPSC-CM transcriptome, respectively (20, 22), comparing favorably but not exceeding the 80% and 65% cellular transcriptome takeover reported after infection of Calu3 human lung epithelial cells and Vero cells, respectively (35, 36). We think that optimal timing of RNA analyses contributed to the superior level of host transcriptome takeover documented here compared to that of Vero and Calu3 cell infections.

As to the differences between the infection levels in the three hiPSC-CM studies, we did select a cardiomyocyte differentiation stage (day 20) with high ACE2 receptor transcript levels, while the ACE2 receptor transcript levels were intermediate in the experiments of Sharma et al. (20), and low in those of Perez-Bermejo et al. (22). We also note that our initial comparative analyses of innate immune responses detected some differences; Sharma et al. and Perez-Bermejo et al. documented induction of certain cytokines during infection, while we did not observe cytokine induction. Interestingly, Sharma et al. but not Perez-Bermejo et al. observed the induction of interferon beta. Thus, differences in the levels of ACE2 expression and in the quality and strength of the innate immune response may contribute to different levels of cellular transcriptome takeover observed in the three studies.

We document here syncytium formation in infected hiPSC-CMs. This phenotype, which is evident in *in vitro* models of human airway epithelium infection (37–40) and was documented also in autopsy material from the respiratory tract of COVID-19 patients (41–43), was not recognized in previously published hiPSC-CM studies (20–24). Higher expression levels of both the ACE2 receptor and the SARS-CoV-2 spike protein in our study compared to other analyses of cardiomyocyte infections may account for more pronounced syncytium formation.

Proper proteolytic activation of the viral spike is required for both cell entry and cell-cell fusion (44). In airway epithelial cells, fusion is triggered by the protease TMPRSS2 and, to a lower extent, by TMPRSS13 (26), which process the spike protein to set free its fusion peptide and elicit membrane fusion. SARS-CoV-2 has evolved a multibasic site at the S1-S2 boundary that allows for proteolytic processing of its spike by furin in the *trans*-Golgi complex of the producer cell, rather than during entry into target cells. In 293T and in Vero cells furin is not absolutely required for cell-cell fusion, while the multibasic site and the concomitant presence of TMPRSS2 sustain this process (45). Since our data indicate that TMPRSS2 and TMPRSS13 are not expressed in cardiomyocytes, another protease may trigger fusion in these cells. Cathepsins are candidates based on the transcriptomics data of the hiPSC-CMs, as are proteases that confer fusion competence to the spike proteins of other coronaviruses (46, 47). We will assess the impact of different protease inhibitors on spike protein induction of cardiomyocyte fusion.

We acknowledge that this study has additional limitations. The most important is that all experiments were performed in tissue culture. Human iPSC-CMs used in our study express cardiac differentiation markers and display spontaneous contractile function. However, our cultures do not include fibroblasts, endothelial cells, and collagen networks, as found in myocardium, a complex multicellular tissue. On the other hand, hiPSC-CMs do form structural precursors of the intercalated discs (ID), cell adhesion structures that connect adjacent cardiomyocytes and support synchronized contraction. Several viruses exploit cell adhesion structures to spread rapidly in tissue (48, 49), and indeed, two viruses that can cause cardiac disease enter cells through the coxsackie and adenovirus receptor (31, 50, 51), which colocalizes with IDs. We are thus assessing whether the ACE2 receptor colocalizes with IDs in cardiac tissue. Current research also includes analyses of the cardiac impact of mouse infection with SARS-CoV-2 MA, a mouse-adapted recombinant virus that can use murine ACE2 for entry into cells (52). Moreover, we are assessing whether spike-elicited membrane fusion impacts the spread of action potentials between cardiomyocytes, perhaps via ID-localized conduits.

Finally, our observation that SARS-CoV-2-induced cardiomyocyte fusion is recapitulated by recombinant spike protein expression alone facilitates the development of therapeutics for COVID-19 induced cardiac disease. In particular, the fusion inhibition activity of peptide-based decanoyl-RVKR-CMK derivatives can be precisely quantified (53, 54) after expression of the spike protein alone, a process that does not require biosafety level 3 precautions. Derivatives of these peptides with enhanced bioavailability could be advanced toward clinical trials as inhibitors of COVID-19-induced cardiac disease.

## MATERIALS AND METHODS

### Spinner culture cardiac differentiation of human-iPSCs.

Obtained under the Mayo Clinic institutional review board (IRB) protocol, patient and control human fibroblast-derived iPSCs were maintained in mTESR1 basal medium with mTESR supplement on plates coated with Geltrex (in Dulbecco’s modified Eagle’s medium [DMEM]/F12 medium). Undifferentiated hiPSCs were transitioned and expanded in suspension/spinner culture in DMEM/F12 plus GlutaMAX, StemPro supplement, bovine serum albumin (BSA), and basic fibroblast growth factor (bFGF) with Rho-associated, coiled-coil containing protein kinase (ROCK) inhibitor Y27632 combined with mTESR1 medium and then chemically differentiated by CHIR (Wnt [Wingless and Int-1] activator) IWP-4 (Wnt inhibitor) into cardiomyocytes (CMs) in RPMI 1640 plus B27 minus insulin supplement as beating aggregates. The detailed spinner culture cardiac differentiation protocol is available from J.W.S. upon request. Differentiated hiPSC-CMs were maintained in Gibco cardiomyocyte maintenance medium and attached to fibronectin-coated glass coverslips. Human H9 embryonic stem cells (WiCell) were chemically differentiated into CMs using an analogous protocol in monolayer culture. EdU (5-ethynyl-2′-deoxyuridine) labeling of growing hiPSC-CMs and detection were done as described by the manufacturer (Thermo-Fisher).

### SARS-CoV-2 infection of hiPSC-CMs.

SARS-CoV-2/UW-001/Human/2020/Wisconsin (UW-001) was isolated from a mild case in February 2020 and passaged in VeroE6 cells expressing TMPRSS2. The virus was used to infect hiPSC-CMs in monolayer at an MOI of 1.0 to 0.001 for 30 min at 37°C. Unbound virus was then washed off, and replaced with fresh medium. At the various time points, cells were fixed or extracted, and samples were collected. An MOI of 0.01 for 24 to 48 h proved optimal for observing early stages of SARS-CoV-2 infection in hiPSC-CMs. Beyond 72 h, even at a low starting MOI, cytopathic lysis overwhelmed hiPSC-CM cultures. Highly permissive SARS-CoV-2 infection was observed in 3 different, equivalently differentiated hiPSC-CMs from unrelated donors.

### Virus titration.

The titer of SARS-CoV-2 infectious virus produced by hiPSC-CM was determined by plaque-forming assay done in confluent Vero E6/TMPRSS2 cells. Then, 12-well tissue culture plates were infected with supernatant (undiluted and 10-fold dilutions from 10 to 10^5^) for 30 min at 37°C. After initial exposure, the Vero/TMPRSS2 cells were washed three times to remove unbound virus, and the medium was replaced with 1.0% methylcellulose medium. After an incubation of 3 days at 37°C, the cells were fixed and stained with crystal violet solution, and the plaques were counted to determine PFU/ml.

### RNA sequencing.

The hiPSC CMs were infected with SARS-CoV-2/UW001/Human/2020/Wisconsin (UW-001) at an MOI of 0.01. Cells were lysed in TRIzol and were kept at −80°C. Total RNA of the lysate was extracted using a Direct-zol RNA miniprep kit (R2050). Library preparation and sequencing were performed at the Mayo Clinic Genome Analysis Core (GAC).

Briefly, cDNA libraries were prepared using 100 ng of total RNA according to the manufacturer’s instructions for the Illumina TruSeq stranded total RNA sample prep kit with Ribo-Zero Gold (Illumina, San Diego, CA). The concentration and size distribution of the completed libraries were determined using an Agilent Bioanalyzer DNA 1000 chip (Santa Clara, CA) and Qubit fluorometry (Invitrogen, Carlsbad, CA). Libraries were sequenced at three samples per lane to generate approximately 119 to 137 million fragment reads per sample following Illumina’s standard protocol using the Illumina cBot and HiSeq 3000/4000 PE cluster kit. The flow cells were sequenced as 100 × 2 paired-end reads on an Illumina HiSeq 4000 instrument using a HiSeq 3000/4000 sequencing kit and HD 3.4.0.38 collection software. Base-calling was performed using Illumina’s RTA v2.7.7.

### Bioinformatics and data analysis.

The quality of the raw RNA-seq data was assessed using fastqp v0.20.1 (55), and quality reads were filtered and aligned against human genome (hg19) using STAR alignment (v2.7.8a) (56) in the Galaxy platform (https://usegalaxy.org). The aligned reads were counted using HTSeq-count v0.9.1 (57), and 0.5 read counts per million (CPM) in at least two samples was used as an expression threshold. Trimmed mean of M (TMM) values normalized (58) and log_2_ transformed data were used for plotting heatmaps and differential analysis in limma (59). For the detection of viral transcripts, quality-filtered reads were aligned against a SARS-CoV-2 genome (GenBank accession number MT039887.1) using the Burrows-Wheeler Aligner MEM algorithm (BWA-MEM) v0.7.17.1 (https://arxiv.org/abs/1303.3997). Alignment summary statistics were computed using SAMtools idxstats v2.0.3 (60). The same workflow was used to reanalyze the RNA-seq data from references 20 (Gene Expression Onmibus [GEO] number GSE150392) and 22 (GEO number GSE156754), except for SARS-CoV-2 genome (GenBank accession number MN985325.1). The raw RNA-seq data from this study are available at the Gene Expression Omnibus (https://www.ncbi.nlm.nih.gov/geo/query/acc.cgi) under accession number GSE184715.

### Immunocytochemistry.

Coverslips were fixed with neutral buffered formalin for 15 min at room temperature, washed with phosphate-buffered saline (PBS)/0.05% Tween 20 and blocked in PBS/5% normal goat serum or 3% BSA/0.3% Triton X-100 at room temperature for 1 h. Coverslips were incubated in primary antibodies diluted in PBS/1% BSA/0.3% Triton X-100 overnight at 4°C, washed extensively, incubated with diluted secondary antibodies (1:400) at room temperature for 1 h, then DAPI stained for 10 min at room temperature. Coverslips were mounted on slides with Prolong Gold antifade mountant (Thermo Fisher) and stored at 4°C. Coverslips were imaged using a Zeiss LSM780 or Elyra PS.1 Super Resolution confocal microscope. Antibodies and reagents for immunocytochemistry included ACTC1 (actin α-sarcomeric mouse monoclonal antibody [MAb] clone 5C5 [Sigma]), phalloidin Alexa Fluor-568 conjugated (Invitrogen), SARS-CoV-2 spike MAb clone 1A9 (GeneTex), SARS-CoV-2 M rabbit polyclonal Ab (Argio Biolaboratories), SARS-CoV-2 nucleocapsid clone 1C7 (Bioss Antibodies), ACE2 goat polyclonal Ab (R&D Systems), and ATP2A2/SERCA2 rabbit polyclonal Ab (Cell Signaling).

### Transmission electron microscopy.

Cells were washed with PBS and placed in Trump’s universal electron microscopy (EM) fixative (61) (4% formaldehyde, 1% glutaraldehyde in 0.1 M phosphate buffer, pH 7.2) for 1 h or longer at 4°C. After 2 rinses in 0.1 M sodium phosphate buffer (pH 7.2), samples were placed in 1% osmium tetroxide in the same buffer for 1 h at room temperature. Samples were rinsed 2 times in distilled water and dehydrated in an ethanolic series culminating in two changes of 100% acetone. Tissues were then placed in a mixture of Epon/Araldite epoxy resin and acetone (1:1) for 30 min, followed by 2 h in 100% resin with 2 changes. Finally, samples were placed in fresh 100% resin polymerized at 65°C for 12 h or longer. Ultrathin (70 to 90 nm) sections were cut with a diamond knife and stained with lead citrate. Images were captured with a Gatan digital camera on a JEOL 1400 plus transmission electron microscope operated at 80 KeV.

### Scanning electron microscopy.

Fixed in Trump’s EM fixative (1% glutaraldehyde and 4% formaldehyde in 0.1 M phosphate buffer, pH 7.2) (61), tissue was then rinsed for 30 min in 2 changes of 0.1 M phosphate buffer, pH 7.2. Following dehydration in progressive concentrations of ethanol to 100%, the samples were critical-point dried. Specimens were then mounted on aluminum stubs and sputter coated with gold/palladium. Images were captured on a Hitachi S4700 scanning electron microscope operating at 3 kV.

### HeLa and Vero cells.

HeLa cells were cultured in Dulbecco’s modified Eagle’s medium (DMEM) supplemented with 10% fetal bovine serum (FBS). Vero-hSLAM (Vero cells stably expressing human signaling lymphocyte activation molecules, kindly provided by Y. Yanagi; these cells are described simply as Vero cells in the manuscript) (62) were maintained in DMEM supplemented with 10% FBS and 0.5 mg of G418/ml. All cell lines were incubated at 37°C with 5% CO_2_.

### Plasmids and mutagenesis.

The codon-optimized SARS-CoV-2 S-protein gene (GenBank accession number YP_009724390) was synthesized by Genewiz in a pUC57-Amp plasmid (kindly provided by M. Barry). The S-protein coding sequence was cloned into a pCG mammalian expression plasmid (63) using unique restriction sites BamHI and SpeI. The SARS CoV S-protein (VG40150-G-N) and the MERS S-protein (C-terminal FLAG tag, VG40069-CF) purchased from Sino Biological, were cloned into the pCG vector for comparative studies. The SARS-CoV-2 S-mEmerald construct was made by cloning the mEmerald sequence (Addgene, plasmid no. 53976) to the C-terminal end of the SARS CoV-2 S-protein in the pCG expression vector. A flexible 6-amino acid-linker (TSGTGG) was used to space the two proteins. All expression constructs were verified by sequencing the entire coding region. The R682S furin cleavage mutation was introduced into the SARS-CoV-2 S expression plasmid by QuikChange site-directed mutagenesis (Agilent Technologies, Santa Clara, CA) according to the manufacturer’s instructions. The clones were verified by sequencing the S-protein gene in the vicinity of the mutation. Two independent clones were tested.

### Immunoblots.

Vero cells were transfected with spike protein expression constructs using the GeneJuice transfection reagent (Novagen). The indicated S-protein expression constructs (1 μg) were transfected into 2.5 × 10^5^ Vero cells in 12-well plates. Then, 36 h posttransfection, extracts were prepared using cell lysis buffer (Cell Signaling Technology, no. 9803) supplemented with cOmplete protease inhibitor cocktail (Roche, Basel, Switzerland), and the proteins were separated by sodium dodecyl sulfate-polyacrylamide gel electrophoresis (SDS-PAGE) (4 to 15% gradient) under reducing conditions. For hiPSC-CMs transfected with CoV-2 S (2 μg/well in 6-well plates), extracts were prepared in cell lysis buffer as described above (but also including phenylmethylsulfonyl fluoride [PMSF]) and separated by SDS-PAGE under reducing (β-mercaptoethanol) or nonreducing conditions. The S-proteins were visualized on an immunoblot using the anti-S specific monoclonal antibody 1A9 (GeneTex, GTX632604; 1:2,000 dilution), which binds the S2 subunit of SARS CoV and SARS-CoV-2 S-proteins. An anti-mouse horseradish peroxidase (HRP)-conjugated secondary antibody was used to reveal the bands. MERS S-protein was detected using a monoclonal anti-FLAG M2-HRP-conjugated antibody (Sigma, A8592; at 1:2,000) which bound to a C-terminal FLAG-tag. The expression of the mEmerald tag was verified using a polyclonal anti-green fluorescent protein GFP antibody (Abcam, ab290; at 1:5,000). For hiPSC-CMs infected with SARS-CoV-2 (MOI, 0.01; 48 h), extracts were prepared in cell lysis buffer as described above (but also including PMSF), separated by SDS-PAGE and blotted with S, M, and N antibodies as described in “Immunohistochemistry,” above.

### Cell-cell fusion assays.

For spike glycoprotein-mediated cell-to-cell fusion, 1.5 × 10^5^ Vero cells in 24-well plates were transfected with 0.5 μg of the indicated S-protein expression vector using the GeneJuice transfection reagent (Novagen) and syncytium formation monitored for 24 to 48 h posttransfection. Images were collected with a Eclipse TE300 microscope using NIS-Elements F 3.0 software (Nikon Instruments, Melville, NY, USA). For recombinant spike glycoprotein-mediated fusion in hiPSC-CMs, subconfluent day-20 differentiated cells plated on fibronectin-coated glass coverslips in 6-well plates were transfected with 1 to 2 μg plasmid using Lipofectamine 3000. For CoV-2 S-mEm in hiPSC-CM experiments, syncytium formation became obvious within 6 h of transfection.

### Furin inhibitor treatment.

Furin inhibitor I (decanoyl-RVKR-CMK; Calbiochem, no. 344930) dissolved in dimethyl sulfoxide (DMSO) was added to Vero or hiPSC-CM cell culture medium 2 h posttransfection. Cell-cell fusion was followed for 72 h for Vero cells and 5 days for hiPSC-CMs, with medium and inhibitor refreshed on day-3. Whole-cell extracts were separated on SDS-PAGE and immunoblotted for SARS-CoV-2 S as described above, or cells were fixed and stained with crystal violet.

### Quantification of hiPSC-CM fusion.

Quantification of percent nuclei in syncytia for mock- versus SARS-CoV-2-infected cells was done as follows. Following immunocytochemistry as previously described, mock-infected and SARS-CoV-2-infected hiPSC-CMs were imaged on a Zeiss LSM780 confocal microscope at ×20 magnification. DAPI-stained nuclei within alpha-actinin stained cell bodies were counted manually using Zen 3.2 Blue software. Then, 12 images from 3 biological replicates were counted for each condition, with an average of 44 nuclei per image. Syncytia were defined as cell bodies containing 3 or more nuclei. Percent nuclei within a syncytium denotes the percentage of total nuclei counted within a syncytium at 48 h postinfection.

Quantification of percent nuclei in syncytia for SARS-CoV-2 spike transfected cells was done as follows. Human iPSC-CMs were plated on 35-mm round glass-bottom dishes and transfected with SARS-CoV-2 spike protein, with or without furin inhibitor treatment, or transfected with SARS-CoV-2 R682S mutant spike protein, as previously described. Bright-field microscopy images were taken at ×10 magnification from randomly chosen areas of each culture dish. Five images from three independent replicates were counted for each condition. Images were manually counted for number of nuclei, number of syncytia, and number of nuclei per cell using Olympus Dimension cellSens software. Percent nuclei within a syncytium denotes the percentage of total nuclei counted within a syncytium at 48 h posttransfection. Syncytia are defined by a cell containing three or more nuclei. Analysis of variance (ANOVA) statistical analysis was carried out using GraphPad Prism software.

### Fluorescence-activated cell sorting.

To determine S-protein cell surface expression levels, HeLa cells (8 × 10^5^ in a 6-well plate) were transfected with the indicated S-protein expression plasmids (2 μg using GeneJuice transfection reagent). Then, 36 h posttransfection, cells were washed in PBS and detached by incubating with Versene (Life Technologies) at 37°C for 10 min. The resuspended cells were washed twice with cold fluorescence-activated cell sorter (FACS) wash buffer (phosphate-buffered saline, 2% FBS, 0.1% sodium azide) and then incubated with the anti-S-protein MAb 1A9 (GeneTex; 1:50 dilution) for 1 h on ice. Cells were washed three times with cold FACS wash buffer and incubated with an AF647-conjugated secondary antibody (Thermo Fisher Scientific, a21235; at 1:200) for 1 h on ice. After three washes with FACS wash buffer, cells were fixed in 4% paraformaldehyde and analyzed with a FACSCalibur (BD Biosciences, San Jose, CA) cytometer and FlowJo software (Tree Star, Inc., Ashland, OR).

### Data availability.

The raw RNA-Seq data from this study are available at Gene Expression Omnibus (https://www.ncbi.nlm.nih.gov/geo/query/acc.cgi) under accession number GSE184715.
