# Supplementary material for: Highly Efficient SARS-CoV-2 Infection of Human Cardiomyocytes: Spike Protein-Mediated Cell Fusion and Its Inhibition
Source: J Virol. 2021 Nov 23;95(24):e01368-21. doi: 10.1128/JVI.01368-21 (PMC8610601; doi:10.1128/JVI.01368-21)
Supplement: Supplemental file 1 — Table S1. Download jvi.01368-21-s0001.pdf, PDF file, 0.2 MB [file jvi.01368-21-s0001.pdf]

# Supporting information

Affymetrix microarray analyses of ACE2 and TMPRSS2 expression in H9 human embryonic stem cells.

**S1 Table.** ACE2 and TMPRSS2 expression in H9 human embryonic stem cells.

| Probe set | Day 0 | Day 8 | Day 20 | Day 50 |
|---|---|---|---|---|
| *ACE2* 219962_at | 11.83 P[*,+] | 4.53 P | 462.73 P | 82.48 P |
| *ACE2* 222257_s_at | 6.61 A[$] | 16.74 P | 672.49 P | 119.02 P |
| *TMPRSS2* 1570433_at | 9.75 A | 15.73 A | 11.82 A | 17.11 A |
| *TMPRSS2* 205102_at | 79.07 A | 47.56 A | 104.44 A | 80.07 A |
| *CTSB* 213275_x_at | 355.25 P | 1330.85 P | 1841.63 P | 1599.04 P |

* Affymetrix microarray numerical values across an individual probe set

[+] P (present): transcript is significantly (P <0.05) expressed compared with perfectly matched and mismatched (background) probe sets

[$] A (absent): transcript is not significantly (P >0.05) expressed
